# 

**Published:** 1867-12

**Authors:** 


### BIBLIOGRAPHICAL NOTICES.

---

*The Physician's Hand Book for* 1868. *By William Elmer, M. D. W. A. Townsend and Adams, publishers, New York.* Contents.—Index to Diseases, Classification of Diseases, Ready Methods in Asphyxia, Poisons and Antidotes, Diagnostic Examination of the Urine. Pulse: Table of, etc. List of Incompatibles, Med. weights and Measures, Abbrev. of Med. Prop. of Remedies, Table of Signs and Abbreviations, Materia Medica, Index of Remedial Agents, Art of writing Extemporaneous Prescriptions, Record of Practice, Calender for 1868, Names and Addresses, Bills and accounts, Record of Practice and Treatment, Table of signs for Daily Practice, Obstetrics and Diagnostic Records, Obstetric Calender, General Remarks.

---

*The Humboldt Medical Archives.*—We wrote a notice of this new candidate for professional favour for our last number, but our printer managed to lose it. We therefore now take occasion to say that it is a very handsome periodical, and edited with decided ability. Its editorial corps is made up of the faculty of the Humboldt Medical College. Its contents are interesting and varied, and the journal is in every way worthy the favourable consideration of the profession.

